# An optimized Protocol for Human M2 Macrophages using M-CSF and IL-4/IL-10/TGF-*β* Yields a Dominant Immunosuppressive Phenotype

**DOI:** 10.1111/sji.12162

**Published:** 2014-04-20

**Authors:** S Mia, A Warnecke, X-M Zhang, V Malmström, R A Harris

**Affiliations:** * Applied Immunology & Immunotherapy, Department of Clinical Neuroscience, Center for Molecular Medicine, Karolinska University Hospital at Solna, Karolinska InstitutetStockholm, Sweden; † Rheumatology Unit, Department of Medicine, Center for Molecular Medicine, Karolinska University Hospital at Solna, Karolinska InstitutetStockholm, Sweden

## Abstract

Monocytes are highly abundant circulatory effector cells and play a vital role in driving or resolving inflammatory processes depending on their activation phenotype. We investigated and compared a panel of polarization protocols of blood-derived monocytes to achieve a stable, optimal and effective regimen for *in vitro* induction of immunosuppressive human macrophages, evaluating their surface receptor expression, cytokine profile, scavenging function and ability to suppress T-cell proliferation. Importantly, we assessed the effect of copolarization or secondary pro-inflammatory stimulation of a primary anti-inflammatory activation phenotype. A combination of IL-4/IL-10/TGF-*β* yielded a relatively stable and dominant immunosuppressive phenotype characterized by higher IL-10 production and down-regulated TNF-*α*, IL-6, CD86, CD274 and MHC II expression. Functionally, IL-4/IL-10/TGF-*β*-stimulated macrophages (M2) had a potent deactivating effect on a subsequent pro-inflammatory LPS/IFN*γ*-activated macrophage (M1) stimulation and significantly suppressed T-cell proliferation. Monocytes derived from patients with chronic inflammatory diseases could be induced to be anti-inflammatory using this protocol. Pre-differentiation with GM-CSF or M-CSF was further demonstrated to enhance final M1/M2 activation status. Our findings indicate a robust polarization protocol for generation of specific immunosuppressive human monocyte-derived macrophages.

## Introduction

Monocytes are highly dynamic and versatile mononuclear phagocytes and are involved in steady-state homeostasis, innate immune surveillance, establishment and resolution in inflammation 1. When appropriately activated, monocytes conduct context-dependent functions in inflammatory, antimicrobial or antitumour responses 2, the local environment phenotypically polarizing the monocytes into macrophages to enable these specific functions 3. Distinct murine subsets (Ly6C^high^/Ly6C^low^) have specific roles in these processes 4, the spleen being a major reservoir of undifferentiated Ly6C^high^ monocytes that are readily mobilized during inflammatory processes 5. In contrast to blood monocytes, major tissue resident macrophage populations are now understood to originate from the yolk sac or foetal liver 6,7, to proliferate locally 8,9 and to transiently relocate early during focal inflammation 10.

Plasticity and diversity are the hallmarks of macrophage lineages. Two major extreme activation phenotypes have been described in both rodents and humans, represented by M1 (classical or pro-inflammatory) and M2 (alternative or anti–inflammatory). It has become increasingly clear that there is an overlapping spectrum of these activation phenotypes 11. We have previously compared induced macrophage activation phenotypes from autoimmune-resistant and autoimmune-susceptible rodent strains and have determined that autoimmune-susceptible strains have a common pro-inflammatory phenotype that leads to perpetuation of inflammation instead of its resolution 12. Similar genetically determined aberrant macrophage phenotypes have also been reported for Type 1 diabetic NOD mice 13. Taken together, this implies that there is a genetic predisposition for autoimmune susceptibility linked to monocyte-macrophage functionality.

The outcome of a focal inflammatory response will be the net result of the relative functions, activation states and numbers of differentially activated infiltrating and resident myeloid cells under the auspices of genetic influences and local environmental cues. In states of chronic inflammation such as autoimmune diseases, there is an apparent imbalance in the pro-inflammatory (pathogenic) and anti-inflammatory (resolution) properties of myeloid cells.

Macrophage colony-stimulating factor (M-CSF) and granulocyte-macrophage colony-stimulating factor (GM-CSF) are not only important hematopoietic growth factors but also potent cytokines that are needed for cell survival, proliferation, differentiation and activation 14. While circulating monocytes are exposed to M-CSF in the blood, exposure to GM-CSF will occur primarily in tissues upon their infiltration into sites of focal inflammation. Functional heterogeneity induced by colony-stimulating factors in monocyte-derived macrophages is well-documented 15, GM-CSF- and M-CSF-derived macrophages being hypothesized to be polarized to M1 and M2 states, respectively 16–18.

Gene expression profiling studies have reported important differences between human and mouse monocyte subsets 19, between different human monocyte subsets 20,21, and distinct genetic fingerprints have even been reported for single human monocyte subsets 22. We previously defined a protocol for induction of potent anti-inflammatory macrophages in mice 23. The purpose of the current study was thus to refine this protocol to induce potent anti-inflammatory macrophages from human monocytes.

## Materials and methods

### Reagents

Lipopolysaccharide (LPS) (Sigma, St. Louise, MO, USA), M-CSF and GM-CSF (PeproTech, Rocky Hill, NJ, USA) were applied at final concentrations of 50 ng/ml. Recombinant human cytokines IL-4, IL-10, IL-13, IFN*γ* and TGF-*β* (R&D Systems, Minneapolis, MN, USA) were applied at final concentrations of 20 ng/ml. Antibodies against CD86, CD273, CD274, CD14, CD206 (all BD Bioscience-Pharmingen, San Diego, CA, USA), HLA-DR (eBioscience, San Diego, CA, USA) and isotype control mouse IgG2a (BD Bioscience-Pharmingen and eBioscience), mouse IgG2b (BD Bioscience-Pharmingen), were used for flow cytometric analyses. Dextran Alexa Fluor 647, 10,000 MW, anionic (Life Technologies, Stockholm, Sweden) was used for endocytosis experiments at 10 *μ*g/ml.

### Monocyte isolation and purification

Human monocytes were isolated and purified from peripheral blood mononuclear cells (PBMC) of buffy coats obtained from healthy volunteers (Clinical Immunology and Transfusion Medicine Department of Karolinska Institutet, Sweden) using Ficoll-Hypaque (GE Healthcare, Uppsala, Sweden) according to the manufacturer's instructions. After isolation from the PBMC using a CD14^+^ selection kit (Miltenyi, Biotech, GmBH, Germany), monocytes were cultured at a concentration of 2 × 10^5^/ml in RPMI 1640 (Gibco, Grand Island, NY, USA), supplemented with 10% heat-inactivated foetal bovine serum (FCS; Sigma), 100 U/ml penicillin, 100 *μ*g/ml streptomycin, 2 mm l-glutamine and 20 μM *β*-mercaptoethanol (all reagents from Life Technologies, Stockholm, Sweden). A total of 25 independent donors were used with minimal variation observed, and for each analysis, at least four different donors are represented.

Peripheral blood mononuclear cells were also obtained from 12 relapsing-remitting multiple sclerosis patients (seven males and five females with median age 39 years) from the in-house biobank of the Neurology Clinic of Karolinska Hospital, Sweden. Blood samples of nine spondylarthritis (SpA) (six female and three male with median age 43 years) were collected from the Rheumatology Clinic of Karolinska Hospital, Sweden. All patients were receiving medication and gave their informed consent, and the studies were approved by the local ethical committee.

### Monocyte polarization, differentiation and activation

Overnight-plated monocytes were polarized for 24 h to M1 macrophages using LPS and IFN*γ* and to M2 macrophages by different combinations of IL-4, IL-10, IL-13 and TGF-*β*, or left untreated for the duration of the culture (M0). The cytokine doses were 20 ng/ml and LPS was used at 50 ng/ml. Monocytes were also pre-differentiated into macrophages by culture for 6 days in RPMI/10% FCS supplemented with 50 ng/ml of either M-CSF or GM-CSF in 6-well culture plates at a cell concentration of 2 × 10^6^/ml.

### Flow cytometry

Twenty-four hour-stimulated monocytes were stained with a cocktail of mAbs (PE-CD163, FITC- CD80, PerCP-Cy™ 5.5-CD86, APC-CD206, PE-Cy™ 7-CD274, and Pacific blue-HLA-DR) and dead cell markers (Life Technologies) with appropriate isotype control stainings. Samples were acquired with a Gallios flow cytometer (Beckman Coulter, Brea, CA, USA) and analysed using Kaluza v1.1 software (Beckman Coulter). Expression was quantified using median fluorescence intensity (MFI) of the marker of interest. Isotype controls were applied to control for background signals.

### Endocytosis assay

Endocytosis of polarized monocytes was measured by cellular uptake of 10 *μ*g/ml Dextran Alexa Fluor 647 (Life Technologies) for 2 h at 37 °C and quantified by flow cytometry. Propodium iodide was used to exclude dead cells. MFI of Alexa-dextran was quantified by flow cytometry.

### ELISA cytokine analysis

ELISA kits for detection of secreted TNF-*α*, IL-6, TGF-*β* and IL-10 in cell culture supernatants were purchased from R&D Systems and used according to the manufacturer's instructions.

### T-cell suppression assay

Optimization of the ratio between macrophages and PBMC determined that a 1:4–1:8 macrophage/ PBMC ratio was effective for T-cell suppression. For the assay, 25 × 10^3^/well macrophages and 10–20 × 10^4^ autologous PBMC were incubated for 72 h in a total volume of 200 *μ*l of complete media (RPMI+FCS) with 1 *μ*g/ml anti-CD3 (OKT3). Cells were pulsed with 1 *μ*Ci/well [methyl-^3^H] thymidine (Amersham, Aylesbury, UK) for 18 h before harvesting and analysis using a liquid *β*-scintillation counter.

### RNA isolation, cDNA synthesis and RT-PCR

RNA was isolated from 1 × 10^6^ cells/ml stimulated monocytes and RT-PCR performed as described 24. Data were analysed using Bio-Rad's CFX Manager 2.0, and several housekeeping genes (i.e. GAPDH, HPRT, *β*-actin and 18S) were used for normalization. All experiments were related to untreated control. Primer sequences are listed in Supplementary Fig. 2. Heatmaps were generated in R (http://www.r-project.org/) using donor median values. Rows and columns were subjected to unsupervised clustering using the distance function: 1-cor(t(x)) applying Spearman's correlation. The colour gradients are scaled for each gene.

### Statistical analysis

Statistical significance was determined by Mann–Whitney *U*-test unless stated otherwise. PCR data were analysed using one-way ANOVA with Tukey's multiple comparison correction. A *P* < 0.05 (*) was considered significant. Statistical analysis was conducted using GraphPad software (San Diego, CA, USA).

## Results

### Monocyte polarization with IL-4/IL-10/TGF-*β* induces a unique suppressive M2 macrophage phenotype

**Figure 1:**
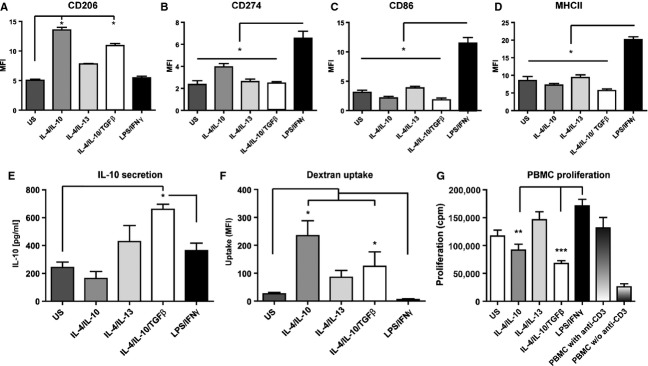
*In vitro* characterization of polarized monocytes. Levels of (A) CD206, (B) CD274, (C) CD86 and (D) MHC II receptor expression analysed by flow cytometry (FACS) following polarization of cells as indicated for 24 h. Results are representative of two separate experiments using different donors, and FACS plots are depicted in Figure S1. (E) IL-10 secretion was measured following stimulation of 2 × 10^5^ monocytes for 2 h followed by washing and further incubation for 24 h. (F) Endocytosis was assessed by stimulating 0.5 × 10^6^ monocytes for 24 h with the respective polarizing agents followed by 4 h incubation with Alexa Fluor 647-coupled dextran. Bars represent mean fluorescence intensity. Results are representative of two separate experiments using different donors. Statistical comparisons were made against untreated (dark grey bars) and LPS/IFN*γ* controls (black bars; *n* = 4/group). (G) T-cell proliferation was determined by polarizing 2.5 × 10^4^ monocytes by the described combinations for 24 h, followed by washing and further coculture with PBMCs and *α*CD3 antibody for 72 h. Results are representative of three separate experiments using four independent donors. Statistical comparisons were made as indicated. Significance levels: *P < 0.05, **P < 0.01, ***P < 0.001.

We first screened phenotypes of healthy blood donor-derived monocytes with a panel of polarization protocols. We used a combination of LPS/IFN*γ* as a potent M1 activation protocol, IL-4/IL-13 as a wound-healing M2 activation protocol and IL-4/IL-10 with or without TGF*β* as deactivating M2 protocols. We assessed expression of surface receptors including costimulatory receptors (CD80, CD86, CD273 and CD274) together with MHC II and CD206 (mannose receptor). Our results revealed that CD206 was more highly expressed in M2 cells activated by IL-4/IL-10 or IL-4/IL-10/TGF-*β* (Fig.1A), while CD274 (Fig.1B), CD86 (Fig.1C) and MHC II (Fig.1D) were expressed at significantly higher levels in LPS/IFN*γ*-stimulated cells than in M2 cells. The polarization protocols thus induce distinguishable macrophage phenotypes from human monocytes.

As IL-10 is a potent anti-inflammatory cytokine and plays a vital role in resolving inflammation, we investigated whether any of the M2 induction protocols had the ability to induce IL-10 secretion. The combination of IL-4/IL-10/TGF-*β* induced significantly higher IL-10 secretion (Fig.1E).

Endocytosis is involved in many important cellular functions during inflammation, including clearance of apoptotic cells and cellular debris from the local environment as well as antigen presentation. We therefore investigated how the panel of stimulants affected the endocytic properties of monocytes by measuring uptake of fluorescent dextran. The results clearly demonstrate that the combination of IL-4/IL-10 or IL-4/IL-10/TGF-*β* induces endocytic activity (Fig.1F).

Suppression of T-cell proliferation has been reported as a key feature of M2 cells in both tumour and inflammation biology settings. To address the suppressive ability of the M2 monocytes, we cocultured them with anti-CD3-activated PBMC. M2 monocytes stimulated with IL-4/IL-10/TGF-*β* significantly suppressed T-cell proliferation compared to M1 monocytes (LPS/IFN*γ* stimulated) that potentiated proliferation (Fig.1G).

Taken together, these data indicate that the same cytokine combination (IL-4/IL-10/TGF-*β*) that was previously determined to be optimal for mouse macrophages could also induce human monocytes to adopt a potent anti-inflammatory M2 macrophage phenotype.

### Hierarchy of macrophage activation states

We next investigated the hierarchy of M1/M2 activations through firstly copolarizing monocytes with both protocols simultaneously and then using sequential M2/M1 stimulations. We specifically addressed the net effects on pro-inflammatory M1 pathways in both of these experimental settings.

**Figure 2:**
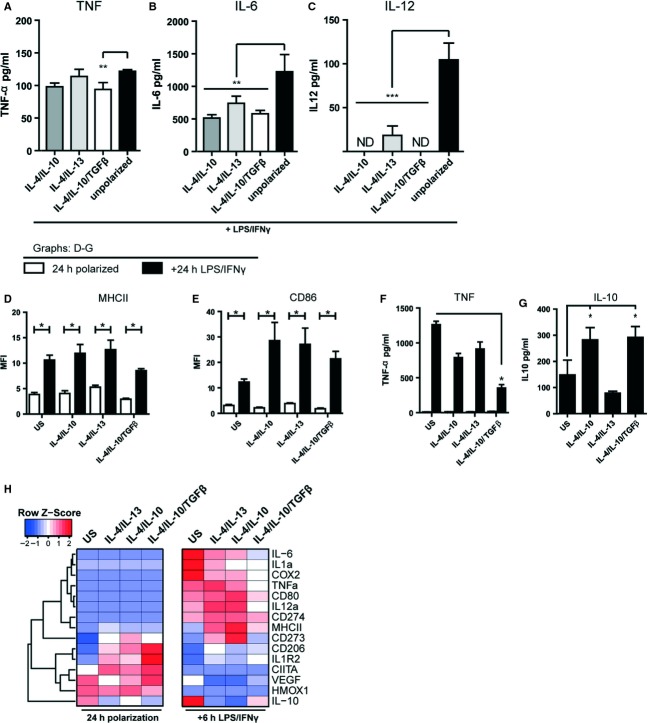
The M2 macrophage phenotype is relatively stable. The relative potencies of M1 and M2 activations were assessed by applying both polarization protocols simultaneously to 2 × 10^5^ monocytes. All bars represent stimulation with LPS/IFN*γ* and individual cytokine combinations or no stimulation (unpolarized) are indicated for each individual bar. Levels of secreted (A) TNF-α, (B) IL-6 and (C) IL-12 were measured by ELISA. Statistical comparisons were made against the LPS/IFN*γ* controls (black bars, *n* = 4/group). M2 activation phenotype stability was evaluated by polarizing 2 × 10^5^ monocytes with the respective combinations for 24 h, followed by washing and sequential stimulation with LPS/IFN*γ* for 24 h. Surface expression of (D) MHC II and (E) CD86 were assessed by FACS and production of (F) TNF-*α* (24 and 48 h), and (G) IL-10 (48 h) was assessed using ELISA. Results are representative of two separate experiments using independent donors (*n* = 4/group per experiment). Statistical comparisons were made as indicated. (H) Gene expression analysis of selected M1- and M2-associated transcripts in monocyte-derived cells following 24-h polarization and subsequent LPS/IFN*γ* challenge for 6 h. Heatmaps depict relative expression levels. Significance levels: *P < 0.05, **P < 0.01, ***P < 0.001.

In the copolarization setting TNF-*α* (Fig.2A), IL-6 (Fig.2B) and IL-12 (Fig.2C) levels were consistently significantly reduced compared to the LPS/IFN*γ*-stimulated M1 control by the synergistic effects of IL-4/IL-10/TGF-*β*. To determine the relative stability of the M2 phenotype following a secondary pro-inflammatory stimulation, we next first induced monocytes with M2 protocols for 24 h and then after washing stimulated with LPS/IFN*γ* for an additional 24 h. Following this secondary challenge, IL-4/IL-10/TGF-*β*-stimulated cells displayed elevated expression of MHC II (Fig.2D) and CD86 (Fig.2E), but importantly had low levels of TNF-*α* production (Fig.2F) and secreted significantly higher levels of IL-10 (Fig.2G).

These data thus indicate that IL-4/IL-10/TGF-*β* treatment counteracts M1 activation during copolarization and induces a robust M2 phenotype in monocytes that does not appreciably switch to an M1 phenotype following secondary pro-inflammatory stimulation.

### mRNA expression in M2 monocyte-derived cells

To extend the phenotypic analyses, we screened expression of a selected range of M1 and M2 transcriptional (mRNA) markers representative of both activation states following polarization of monocytes for 24 h (Fig2H). The established M1- and M2-associated transcripts faithfully clustered away from each other as judged from the dendrogram (Fig2H, left). The M2-associated markers (represented in the lower half), namely CD206, IL1R2, CIITA, VEGF and HMOX1, were most highly induced following polarization with IL-4/IL-10/TGF-*β*.

To extend this analysis, primarily M2-polarized cells were secondarily challenged with LPS/IFN*γ* (M2→M1, Fig2H right-hand panel). A clear response to M1 stimulation was that M1-associated transcripts (upper half) were highly induced. However, in all M2-polarized cells, the levels of M1 markers were significantly lower, the reduction being most evident with IL-4/IL-10/TGF-*β* polarized cells. Furthermore, M2 markers such as CD206 or IL1R2 were retained highest with IL-4/IL-10/TGF-*β* polarization. Taken together, these data demonstrate firstly that distinct M2 phenotypes are induced depending on the combination of applied cytokines and secondly that IL-4/IL-10/TGF-*β* polarization is relatively resistant to subsequent M1 conversion.

### M2 monocytes are capable of suppressing T cells from individuals with autoimmune diseases

In states of chronic inflammation such as autoimmune diseases, the assumption is that there is a local dominance of pro-inflammatory immune activity over anti-inflammatory activity in the target organs of individuals with disease. One therapeutic possibility could thus be to adoptively transfer autologous, pre-activated, anti-inflammatory macrophages to down-regulate the pro-inflammatory pathogenesis locally, an approach we have successfully employed in different experimental models of autoimmune disease 25,26. A prerequisite for this approach is that it is possible to induce an anti-inflammatory phenotype in the monocytes of individuals with disease. We thus next investigated this possibility in a small cohort of samples from healthy controls or individuals with either multiple sclerosis (MS) or Spondylarthritis (SpA) using M0 (unstimulated), M1 (LPS/IFN*γ*) or M2 (IL-4/IL-10/TGF-*β*) polarization protocols.

**Figure 3:**
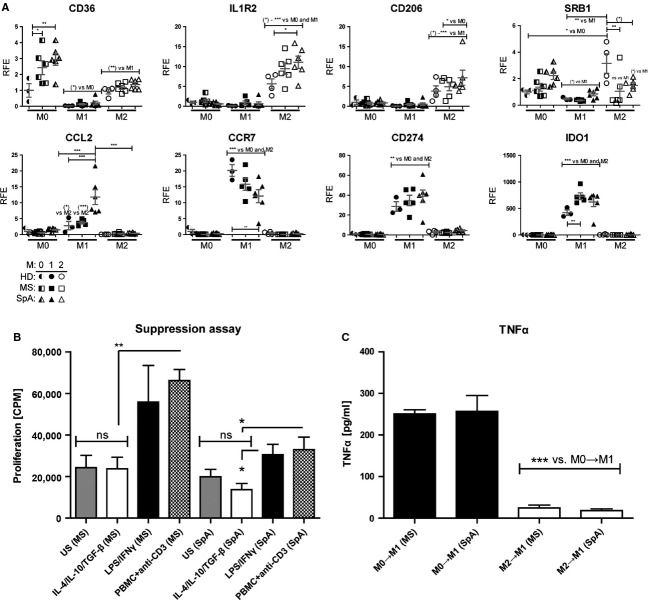
Autologous M2 monocytes are capable of suppressing T cells from individuals with chronic inflammatory diseases. Monocytes were purified from individuals with multiple sclerosis (MS) or spondylarthritis (SpA), or health donors (HD). (A) mRNA expression of selected M1 (LPS/IFN*γ*)- and M2 (IL–4/IL–10/TGF-*β*)- associated transcripts measured after 24 h of polarization. (B) 24-h polarized patient monocyte-derived cells were cocultured with patient PBMCs, and proliferation was assessed as described. (C) ELISA-based detection of TNF-*α* in supernatants of patient monocytes challenged simultaneously with M1 (LPS/IFN*γ*) and M0 (media control) or M2 (IL–4/IL–10/TGF-*β*) protocols, respectively. Significance levels: *P < 0.05, **P < 0.01, ***P < 0.001.

Overall, we observed no consistent defects in the *in vitro* polarization of patient-derived monocytes in the selected targets assessed by RT-PCR as most followed the same trends as in the healthy controls (Fig3A). These data demonstrate that comparable macrophage phenotypes to healthy controls, both M2 anti-inflammatory (upper row) and M1 pro-inflammatory (lower row), can be induced from monocytes from individuals with pro-inflammatory diseases using the employed polarization protocols.

We next assessed whether monocytes isolated from individuals with the chronic inflammatory conditions MS or SpA could be induced by the IL–4/IL–10/TGF-*β* polarization protocol to exert anti-inflammatory effects. Our results demonstrated that IL–4/IL–10/TGF-*β*-activated M2 cells could significantly suppress proliferation of autologous T cells in samples from both types of disease, respectively (Fig.3B). Secondly, we observed a marked suppression of pro-inflammatory TNF-*α* secretion in M2 macrophages following simultaneous M1 activation (Fig.3C). This demonstrates that *in vitro* polarized M2 cells from patients with chronic pro-inflammatory diseases are still capable of being induced to exert immunosuppressive activities. At this point, it is not well studied to what degree there is heterogeneity in monocyte/macrophage phenotypes in healthy populations or to what degree current patient treatments or disease states affect their monocyte/macrophage phenotypes. We did, however, observe modestly elevated CD36 levels in M0 macrophages of both patient groups. In light of the restricted sample size, the results should be regarded as a proof-of-principle. Samples for an extended macrophage activation proteomics study accounting for current disease state, treatment and age in much larger patient cohorts are currently being collected.

### The effect of GM-CSF and M-CSF pre-differentiation on monocyte-derived macrophage activation phenotypes

**Figure 4:**
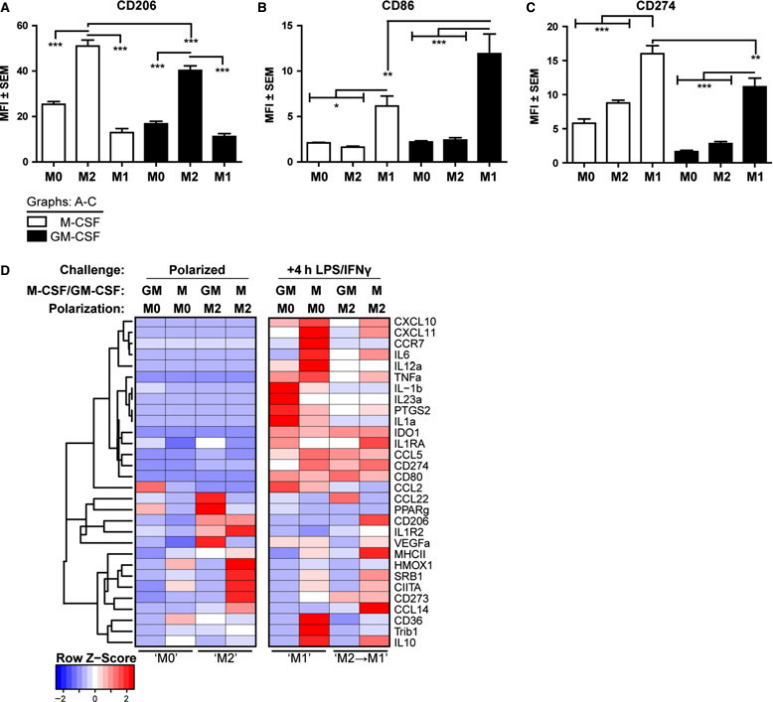
M2 monocyte activation phenotypes following M-CSF or GM-CSF pre-differentiation. 2 × 10^5^ monocytes were differentiated for 6 days with M-CSF or GM-CSF, and cells were further polarized to M0 (no additional stimulation, control), M2 (IL–4/IL–10/TGF-*β*) or M1 (LPS/IFN*γ*) states for 24 h. Levels of (A) CD206, (B) CD86 and (C) CD274 were analysed by flow cytometry. Statistical comparisons were made as indicated. Results are representative of two separate experiments using different donors. (D) An extended cluster analysis of mRNA transcript expression is presented for primary polarization following pre-differentiation (left panel) or following subsequent secondary challenge for 4 h with LPS/IFN*γ* after the initial M0 or M2 polarizations. Individual gene transcript profiles are presented for a panel of previously M1/M2-associated transcripts. The LPS/IFN*γ* challenged M0 groups are representative of M1. Significance levels: *P < 0.05, **P < 0.01, ***P < 0.001.

The bone-marrow-derived mouse macrophages utilized in our previous study were standardly grown in M-CSF during initial differentiation into macrophages. As human monocytes are reported by some authors to represent M1 cells if differentiated with GM-CSF or M2 cells if differentiated with M-CSF, we systematically addressed the phenotypes following (1) only differentiation with each growth factor or (2) combining growth factor-induced differentiation with subsequent specific M1 or M2 activation. Flow cytometric analyses revealed that pre-differentiation with GM-CSF or M-CSF had different effects on resultant macrophage phenotypes. CD206 expression was elevated following M2 activation, with highest expression on M-CSF pre-differentiated macrophages (Fig.4A). CD86 was elevated following M1 activation, with highest expression on GM-CSF pre-differentiated macrophages (Fig.4B). CD274 was elevated following M1 activation, but somewhat surprisingly highest with M-CSF pre-differentiation (Fig.4C). These results indicate that while M1 and M2 activation states can be induced irrespective of pre-differentiation signals, the balance of pre-differentiation growth factors will ultimately define the final level of macrophage activation phenotype.

We also screened the mRNA expression of a selected panel of polarized M1 and M2 cytokines, chemokines and surface receptors following initial M-CSF and GM-CSF pre-differentiation and subsequent polarization (Fig.4D). The left-hand panel of the heatmap depicts primary polarization with IL-4/IL-10/TGF-*β*, and the right-hand panel the subsequent secondary stimulation with LPS/IFN*γ*. The genes cluster into distinct groups, M1 markers located at the top and M2 markers at the bottom, respectively.

Compared to the M0 state, both M-CSF and GM-CSF M2-polarized macrophages expressed M2 gene transcripts, but interestingly the profiles differed. Typical M2 markers CD273, CIITA, SRB1, CD206, IL1R2 and HMOX1 were best induced with M-CSF pre-differentiation (left panel). Following challenge, IL1, IL23 and PTGS2 (COX-2) dominated in GM-CSF pre-differentiated cells, whereas CXCL10, CXCL11, CCR7, IL6, IL12a and TNFa were higher in M-CSF M1 cells. Taken together, these indicate alternative inflammatory responses, which importantly are repressed in M2-preconditioned cells. Furthermore, M-CSF M2 pre-polarized cells expressed higher levels of M2-associated IL10, CCL14 and CD206, which were retained following secondary M1 stimulation (Fig4D, lower part).

In conclusion, these data demonstrated that the most robust IL-4/IL-10/TGF-*β* M2 activation phenotype is induced following initial pre-differentiation with M-CSF.

## Discussion

We conducted the current study to address how best to induce *in vitro* an immunosuppressive human macrophage phenotype. We compared and contrasted selected activation protocols that we have previously tested with mouse cells and concluded that a combination of IL-4/IL-10/TGF*β* yielded a phenotype characterized by: (1) expression of M2-associated surface markers and gene transcripts (e.g. CD206), (2) immunosuppressive cytokines (e.g. IL-10), (3) ability to retain the phenotype on secondary pro-inflammatory activation and (4) ability to suppress autoreactive pathogenic T-lymphocyte proliferation. Moreover, our data clearly indicate that there are both similarities and differences in final monocyte activation states depending on whether M-CSF or GM-CSF is used during pre-differentiation. Following specific subsequent activation, the most potent M2 phenotype was attained in M-CSF pre-differentiated monocytes, while the most potent M1 phenotype was attained in GM-CSF pre-differentiated monocytes, similar to what we have determined with murine bone marrow macrophages. Our conclusion is that we can generate a comparable IL-4/IL-10/TGF-*β*-polarized immunosuppressive macrophage phenotype with respect to both immunological phenotyping and functional activity in both human and murine systems.

Monocytes represent circulating cells that in steady state are exposed to M-CSF in the blood. Given appropriate chemotactic signals, they will infiltrate into tissues, transforming into pro-inflammatory tissue macrophages under the influence of GM-CSF. The tissue-destructive properties of pro-inflammatory M1 macrophages is evident in many settings of chronic inflammatory autoimmune diseases, yet immunosuppressive M2 properties are equally apparent in settings of tumours 27 and helminth infections 28. While significant large numbers of M1 cells are typical of foci of tissue destruction in the former setting, more modest numbers are associated with the latter function. In a given pro-inflammatory focus, the relative balance of M1 cells driving disease and the M2 cells attempting to naturally regulate the immune response will determine the final outcome of either chronic inflammation or healing. Manipulation of this M1/M2 stochastic balance through adoptive transfer of *in vitro* pre-activated macrophages thus represents a relatively logical immunomodulatory strategy and a growing body of researchers explore this possibility 29. How long the transferred cells remain potent in their immunosuppressive functional state requires further investigation. Translation into the clinic has already been attempted, with the first human trials using immunosuppressive macrophages being conducted in a setting of spinal cord injury 30. While the therapeutic effects in animal models of this pathology have been promising, translation into the human setting has been less efficacious.

An important aspect will be stability of the induced anti-inflammatory phenotype, which must not switch on entering a pro-inflammatory focus. Macrophage activation plasticity has been investigated previously, with a first indication of the capability of macrophages to switch phenotype *in vitro* being reported 31. However, another study conversely concluded that the nature of the first of two different consecutively applied stimulants decides the final activation state of the cells 32. Our M2 induction protocol using a combination of IL-4/IL-10/TGF-*β* yielded a phenotype that buffered subsequent pro-inflammatory secondary stimulation. Compared to the strong and persistent challenging stimuli we used *in vitro*, how M2-polarized cells behave in *in vivo* situations remains to be explored.

In clinical settings of pro-inflammatory diseases such as secondary progressive MS or advanced Type 1 diabetes, myeloid cell therapy using anti-inflammatory macrophages is an attractive concept. As the source of macrophages is from the patient themselves there should be ample possibilities for repeated treatments, and this would compensate for the potential short-term immunosuppressive action the transferred cells might have *in vivo*. While a heightened pro-inflammatory monocyte activation has typically been reported from individuals with autoimmune diseases 33–36, our data demonstrate that given an appropriately efficient polarization protocol an effective anti-inflammatory activation state can still be induced in cells recovered from individuals with disease. We consider that the use of stringently optimized polarization protocols to ensure maximal retention of functional immunosuppressive capacity post-transfer is a key consideration for macrophage therapy in pro-inflammatory diseases.
